# Evaluation of Relationships between Menopause Onset Age and Bone Mineral Density and Muscle Strength in Women from South-Western Poland

**DOI:** 10.1155/2020/5410253

**Published:** 2020-06-10

**Authors:** Jarosław Fugiel, Zofia Ignasiak, Anna Skrzek, Teresa Sławińska

**Affiliations:** ^1^Department of Biostructure, University School of Physical Education in Wroclaw, Wroclaw, Poland; ^2^Department of Physiotherapy in Motor Organ Dysfunctions, University School of Physical Education in Wroclaw, Wroclaw, Poland

## Abstract

**Introduction:**

The onset of the menopause entails numerous changes, both physical and mental, in the functioning of the bodies of women. Moreover, the early menopause increases the risk of occurrence of many civilization-related diseases. Major factors contributing to health deficits include lowered bone mineral density and sarcopenia, which can result in serious functional limitations and the acceleration of ageing processes in the body. The aim of this study was to determine how the menopause onset age is linked with bone mineral density and the strength of selected muscles of the limbs and the trunk. *Material and Methods*. 756 women aged 50-80 years were subjected to tests. The subjects were divided into three groups: (I) from 50 to 59 years, (II) from 60 to 69 years, and (III) from 70 to 79 years. Each of the women specified the age when her final menstrual period occurred. On this basis, groups of women with (1) the early menopause—before the 50th year of life—and (2) with the late menopause—after the 50th year of life—were distinguished. Bone mineral density (BMD), dominant hand grip strength, knee extensor and flexor strength, and functional upper and lower body muscle strength were determined in each of the women.

**Results:**

The test results indicate differences in levels of muscle strength and BMD between the 50-year-old early- and late-menopausal women. The late-menopausal women score better motor ability test results and higher BMD values. The differences decrease in the groups of 60-year-old women, whereas the 70-year-old early- and late-menopausal women score similar results.

**Conclusions:**

A higher percentage of women with a lowered bone mass and a lower strength level was found in the group of early-menopausal subjects. The rate of decline in hand grip strength, the functional efficiency of the upper and lower limbs, and BMD is faster in the late-menopausal women, whereby the two groups of 70-year-old women score similar test results.

## 1. Introduction

The menopause is the result of the physiological cessation of the activity of the ovaries. The consequence is not only the termination of the reproductive period, but also numerous changes in the functioning of the woman's body. The early menopause increases the risk of occurrence of many civilization-related diseases [1, 2]. Its symptoms are of both physical and mental nature and vary greatly in the course of the menopause and in the picture of the accompanying changes [3].

It is indicated that many genetic, socioeconomic, environmental, and ethnic factors have a bearing on the menopause onset age [4–6]. Major factors contributing to health deficits due to the reduced activity of the ovaries and the subsequent decline in sex hormones are a reduction in bone mineral density [7] and sarcopenia [8, 9] resulting in serious functional limitations and the accelerated ageing of the body [10].

Bone tissue remodelling in the first two decades of life has a progressive character, reaching the peak of mineral bone mass constituents in the third decade [11]. The peak is higher in men than in women [12]. Researchers are of the opinion that the higher the peak mineral bone mass, the slower its loss in the subsequent decades of life [13] and the later the occurrence of osteopenia and osteoporosis. Among the many health deficits connected with the occurrence of osteoporosis in old age is the compressive fracture of vertebral bodies [14] and the resultant reduction in body height. Changes within the spine lead to body posture deformations [15]. The changes are accompanied by backaches, the lowering of physical fitness and activity, and life quality deterioration. Considering the very complex character of osteoporosis, determined by genetic factors and broadly understood environmental factors, and the slow progress of this disease, further research and in-depth analyses should be conducted to explain which of the factors connected with the dynamically changing environment, including lifestyle, are the primary determinants of osteoporosis. Research results indicate a significant role of BMI (body mass index) in maintaining bone mineral density proper for the given age and sex [16, 17]. A normal body mass or slight overweigh are associated with a higher bone mineral density level. That is why physical exercises in which one's own body mass is employed, i.e., mainly locomotive exercises, such as walking, running, swimming, and cycling, are so important [1, 18].

The average menopause onset age in the countries of Europe falls on the 50-51st year of life [2, 19]. Since the menopause can make professional and social activity difficult, further research and in-depth analyses of the conditions and factors having a mitigating effect on the course of menopause processes are necessary. The aim of this study was to determine how the menopause onset age is linked with bone mineral density and the strength of selected muscles of the limbs and the trunk.

## 2. Material and Methods

### 2.1. Study Design and Settings

The research project was conducted in the form of cross-sectional and exploratory research. It was carried out in the years 2009-2012 as part of the Grant of the Ministry of Science and Higher Education No N N404 075337.

The research was conducted at the Laboratory of Biokinetics of the University, with the exception of the months from November to March. The laboratory holds a Quality Management System Certificate PN-EN ISO 9001:2009 (reg no.: PW-48606-10E).

The project was approved by the Senate Committee on the Ethics of Scientific Research at the University School of Physical Education in Wroclaw (18.02.2009), and the protocol was written in accordance with the standards set by the Declaration of Helsinki. Informed consent was obtained from all individual participants included in the study.

### 2.2. Study Participants and Selection

756 women aged 50-80 years were subjected to tests. The participation in this research project was voluntary, and all the participants were informed that they could withdraw from the tests at any time. The inclusion criteria were as follows: 50 years of age, a written consent for participation in the project, no medical contraindications for participation in the tests, and cessation of menstrual periods. The menopause status was defined on the basis of WHO's definition of the menopause: women with 12-month long or longer period without menstruation which has not ceased due to operation, treatment, pregnancy, breast-feeding, or serious weight loss were defined as naturally postmenopausal [20].

Depending on their age, the subjects were divided into three groups: (I) from 50 to 59 years (*M* = 56.5; SD = 2.3 yrs), (II) from 60 to 69 years (*M* = 63.8; SD = 2.5 yrs), and (III) from 70 to 79 years (*M* = 73.2; SD = 2.6 yrs). Each of the women specified the age of occurrence of her final menstrual period and on this is the basis groups of women with the early menopause—before the 50th year of life—and the late menopause—after the 50th year of life were distinguished. The numbers of participants in groups in the early menopause 50-59 years old (*n* = 90), 60-69 years old (*n* = 216), and 70-79 years old (*n* = 86); and the late menopause 50-59 years old (*n* = 76), 60-69 years old (*n* = 226), and 70-79 years old (*n* = 62).

### 2.3. Research Instruments

Bone mineral density (BMD) was measured in the region of the distal epiphysis of the nondominant limb's forearm. The measurements were performed using peripheral dual-energy X-ray absorptiometry (pDEXA) by means of an EXA-3000 bone densitometer. Bone mineral density was analysed as: BMD (g × cm–2), % peak BMD as *T*-score (%), and % age-specific BMD as *Z*-Score (%).

The dominant hand grip strength (kg) was evaluated using a Jamar 5030J1 SAEHAN hydraulic dynamometer. The strength of the knee extensor and flexor muscles was measured using an Amethyst II multifunctional rehabilitation-diagnostic resistance exercise chair with a force-moment transducer bearing register-of-medicinal-products number PL/DR 011563. The maximum values of the moment of force of the quadriceps (Mmax of quadriceps (Nm)) and the knee flexors (Mmax of knee flexors (Nm)) were analysed. The measurements were performed three times (the best result was taken into account) for the knee joint of the dominant limb in isometric work conditions. Two tests being part of the Senior Fitness Test, the Arm Curl Test and the Chair Stand Test, were used to analyse the level of the functional strength of the upper and lower limbs [21].

#### 2.3.1. Instructions for Testing

*(1) Arm Curl*. For the test procedure, the test person sits on a chair, his back is straight, and his feet are flat on the floor. The 5 lbs weight is held in a more efficient hand with his hand closed. At the start of the test, the arm is facing down next to the chair, perpendicular to the floor. At the start signal, the test person turns his hand upwards, bending his upper limb in the elbow joint, and then straightens it to its initial position. The result is a total number of correctly executed flexions within 30 seconds.

*(2) 30-Second Chair Stand*. For the test procedure, the test person sits on a 43.2 cm high chair with his back straightened out and his feet flat on the floor. The arms are crossed at the wrists and held at the chest. At the start signal, the test person is raised to full standing and then returned to full sitting position. The result is the total number of uprisings and sits properly made in 30 s.

Physical fitness tests were conducted in the morning, in the Biokinetics Laboratory of the University. The measurements were carried out by a team of authors.

The basic somatic features were also measured: height (cm) and body weight (kg), from which the BMI was calculated. Women also provided information on education, smoking and alcohol consumption, and living together or alone.

### 2.4. Data Analysis

The test results were statistically analysed and the basic characteristics were calculated. The normal distribution of variables was assessed using the Shapiro-Wilk test. Average values and standard deviations (SD) were calculated for each test result. The significance of differences between the average results were assessed using a two-factor analysis of variance (ANOVA). Intergroup differences of age and menopause groups were assessed based on the least significant difference test (NIR). In tables, statistically significant values are marked in bold. Statistical significance was adopted at the level of *p* < 0.05. Calculations were made at the Laboratory of Biokinetics of the Department Biostructure at the University School of Physical Education in Wroclaw, using the STATISTICA package version 10.0 (Stat Soft Inc.).

## 3. Results

The women in each of the calendar age groups were divided according to, respectively, the early and late menopause age. Tables 1 and 2 shows the means and standard deviations of the somatic parameters and selected lifestyle factors. The analysis of these parameters showed no differences between women with early and late menopause with the exception of height and weight values for 70-year-old women (Tables 3, 4, and 5).  Table 6 shows the means and standard deviations of the strength and bone mineral density parameters.

The strength levels of the upper limbs, the lower limbs, and the trunk, and bone mineral density were evaluated taking into account two factors: calendar age and menopause onset age. The results of the two-factor analysis of variance confirmed that all the analysed strength and bone mineral density parameters had significantly deteriorated with calendar age (Table 4). In the case of the second considered factor, i.e., menopause age, the significant effect applied mainly to the results of physical fitness functional tests evaluating the strength of the limbs and the trunk. Moreover, the interaction of the two factors also affected the differentiation of upper limb and knee extensor muscle strength (Table 4).

The significant, but less numerous than for calendar age, relationships between menopause onset age and some of the investigated variables were subjected to further detailed analysis.

A comparison between women at the same calendar age, but differing in their menopause age, showed for the group of the youngest women (50-59 yrs) significant differences between the averages for the results of the functional physical fitness tests (the arm curl test and the chair stand test) and the knee extensor strength tests and for the characteristics describing bone mineral density (BMD, *T*-ratio, and *Z*-ratio). In each of the cases, the late-menopausal women scored better results. At the age of 60-69 years, the differences decrease and statistical significance applies only to the large number of arm curl test repetitions in the case of the women in whom the menopause occurred at a later age. In the oldest age category, i.e., 70-79 years, the averages of all the investigated variables are similar and do not differ significantly (Table 5).

**Figure 1:**
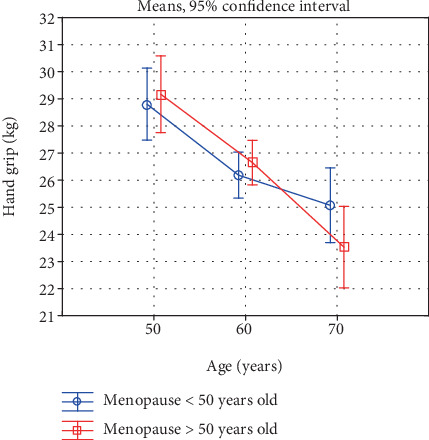
Changes in hand and forearm strength with age in early- and late-menopausal women.

**Figure 2:**
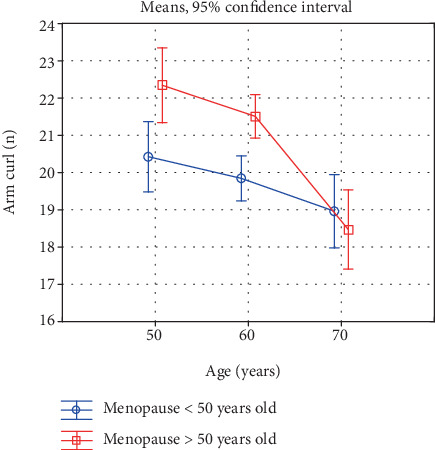
Changes in arm and forearm strength with age in early- and late-menopausal women.

**Figure 3:**
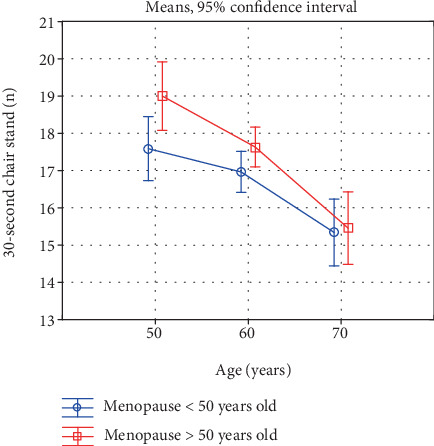
Changes in lower extremities strength with age in early- and late-menopausal women.

**Figure 4:**
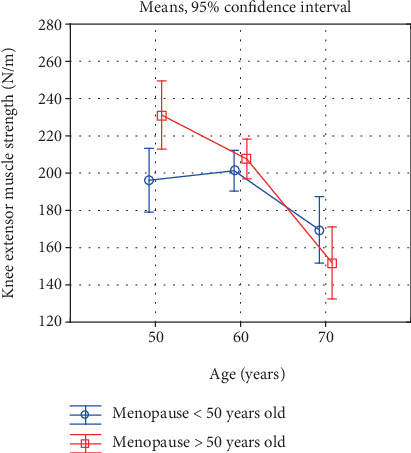
Changes in knee extensor muscle strength with age in early- and late-menopausal women.

**Figure 5:**
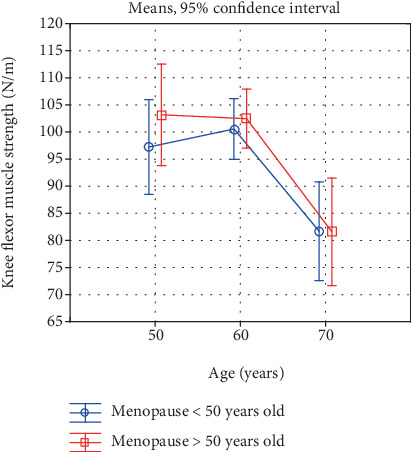
Changes in knee flexor muscle strength with age in early- and late-menopausal women.

**Figure 6:**
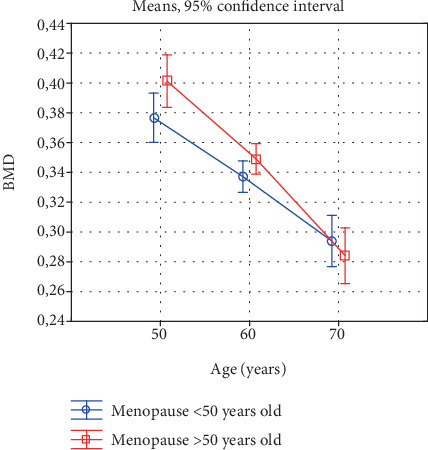
Changes in bone mineral density with age in early- and late-menopausal women.

Also, a comparison of the investigated characteristics in women in the successive age categories, reflecting the rate of the involutional processes, is interesting (Table 5, Figures 1-6). In the early-menopausal women between the 50-59th year of life and the 60-69th year of life, hand and forearm (hand grip) strength and all the analysed BMD parameters significantly decreased. After the 60th year of life, significantly lower test and measurement results were recorded mainly in the case of the lower limb strength parameters. Moreover, the adverse changes in bone mineral density remained at a significant level.

In the late-menopausal women, the rate of the involutional processes was higher. Between the 50-59th year of life and the 60-69th year of life only, the average arm curl test score and the strength of the knee muscles decreased insignificantly. As regards the other analysed strength and bone mineral density characteristics, a significant decline with age was recorded in this period. After the 60th year of life, the rate of the changes continues to be high, whereby the differences between the averages of all the investigated variables are statistically significant.

Due to this characteristic acceleration of the involutional processes affecting appendicular and trunk muscle strength after the 60th year of life in the late-menopausal women, the differences in the averages in the oldest age category decrease and are statistically insignificant. This is illustrated in Figures 1-6.

The decline in bone mineral density with age is significant in both groups of women differing in their menopause onset age. A higher rate of BMD decline was observed in the late-menopausal women (Figure 6). The higher rate reflects the increase in the percentage of women with low bone mass between the consecutive decades. In the early-menopausal women, this is, respectively, 15.4% and 21.7% while in the group of late menopausal women 22.6% and 28.4% (Table 7).

## 4. Discussion

Ageing as a multiaspect and multifactor process is linked with many pathologies affecting different organs, including the skeleton. The age-related loss of bone mass and the resulting osteoporosis expose the elderly population to an increased risk of fractures and the incidence of diseases. Many genetic, hormonal, and biochemical factors are responsible for this phenomenon. Hormonal imbalance, ageing, environmental factors, the style of life, and genetic predispositions are responsible for about 50-80% of the individual bone mineral density loss. The loss of BMD in women consists of two stages. It begins after the menopause, as a quick oestrogen-dependent process with a rapid decrease in bone mass, and lasts about 5-10 years. In this period, about 50% of the total BMD of the thoracic and lumbar spine is lost, which leads to frequent compression fractures of vertebral bodies. After this period, it follows the slow constant age-related loss of bone density, resulting in bone trabeculae rarefaction and the loss of bone tissue in the cortex layer, which increases the susceptibility of the femoral bone neck to fracture. The annual bone mass loss amounts to about 0.5% in premenopausal women, 2-2.5% in women going through the menopause, and about 1.5% in postmenopausal women [22].

The above regularity was confirmed by our investigations. In the tested group of 50-year-old women, a higher percentage of persons with lowered bone mass occurs in the group of early-menopausal women. In the next decades, the percentage of women with a lowered bone mass increases in both the groups, but more in the group of late-menopausal women. Consequently, in the group of 70-year-old women, the percentage of women with a lowered bone mass is similar in the groups of early- and late-menopausal women.

One of the aims of our investigations was to determine the relationship between the menopause onset age and BMD and the functional parameters of the body. In recent years, links between the natural menopause onset age and other than genetic factors, such as the professional status, have been sought. As they have an effect on the autonomic nervous system and neuroendocrinal activity, heavy workloads can affect the reproductive functions. Excessive stress, which increases the stress hormone level, can adversely affect reproductive capacity and accelerate the menopause [2].

For each woman, the early menopause, especially in the course of her professional activity, is a very difficult period. During these years, women experience not only physical changes in their body but also in their psyche, which can affect their professional and family life. Many physicians emphasize that professional activity, performing daily duties, and meeting with friends can mitigate menopause symptoms. The latter can also be reduced through a healthy lifestyle, a healthy diet, regular exercise, maintaining a proper body mass, and preventive measures [23].

The women taking part in our project were subjectively healthy, independent, and physically and socially active. They attended classes organized by senior citizens clubs and the University of the Third Age. They were persons aware of the possibilities of managing the negative effects of the menopause and the need to lead an active healthy lifestyle. Such healthy ageing comprises three main elements: low likelihood of falling ill and of illness-related disability, high cognitive and physical efficiency, and active engagement in life. One of the key elements of healthy ageing is the maintenance of a proper functional efficiency level needed to effectively perform daily activities, such as personal care, housework, and doing shopping. Rikli and Jones [21] developed a well-proven battery of tests dedicated to older persons, mainly the Senior Fitness Test (SFT). The latter was also used in our study. SFT covers aspects of aerobic efficiency, upper and lower body strength, the range of motion, and balance and coordination, which are necessary for the performance of daily activities. The advantage of such tests is the objective measurement of functional fitness. A high level of physical activity improves aerobic efficiency, strengthens the muscles, reduces fatty tissue, and slows down BMD loss. As a result, it contributes to higher walking efficiency and better balance and a lower risk of fall and fracture. Physical activity also reduces the risk of occurrence of many diseases, such as circulatory system diseases, diabetes, and tumours. It also has a favourable effect on the function of neurotransmitters and brain morphology and lessens cognitive disorders and depression as well as improves self-esteem, helping elderly persons in social activity [10].

Summing up several works on the effects of healthy active lifestyle, one can say that a higher level of physical activity results in greater muscle strength and functional efficiency in elderly women [24, 25].

Our research indicates that in the 50-year-old women, there are differences in the levels of muscle strength and BMD between, respectively, the early- and late-menopausal women. The differences decrease in the groups of 60-year-old women, whereas in the case of the 70-year-old women, the motor ability test results and the BMD values in, respectively, the early- and late-menopausal women are similar. The rate of decline in hand grip strength, in the functional efficiency of the upper and lower limbs and in BMD, is faster in the group of late-menopausal women. Consequently, the test results for the women aged 70-80 years in both the analysed groups (with, respectively, the early and late menopause) are similar. Fistarol et al. [26] found the time which passed from the menopause onset and the body mass index to be the principal factors related to osteoporosis. This confirms that no age, but oestrogen deficiency, is the main cause of this disease.

Many studies have shown that low bone mineral density (BMD) is a strong fracture risk factor, but not the only one. Fracture prevention should concentrate more on falls. In postmenopausal women, simultaneous age-related muscle strength decline and bone mass loss are observed. Long-lasting physical activity, involving resistance exercises, is significantly connected with a higher bone mineral density. It seems probable that a physically active lifestyle helps to maintain muscle strength and delay sarcopenia and, consequently, maintain a proper level of BMD. The risk of fractures is greater among women with a high bone loss index, although changes in BMD explain only 20% of the fractures in elderly persons. Some studies have confirmed the relationship between hand grip strength and fractures. Therefore, it seems that muscle strength and functional efficiency tests should be an additional diagnostic tool in screening for osteoporosis and the risk of falls and fractures [7].

Women with osteoporosis can feel a deterioration in their muscular-skeletal system, which may result in the limitation of their daily activity, pain complaints, and an increase in the incidence of falls and, consequently, an increase in the risk of fractures. Therefore, inexpensive and easy-to-use methods, which will make it possible to administer an early treatment and rehabilitation, and so minimize the consequences of the disease, should be used to identify women with osteoporosis [9].

The effect of physical exercise on BMD has been evaluated many times in cross-sectional studies of young and older persons. However, this effect varies greatly depending on age, sex, and the level, kind, and duration of sporting activity. Therefore, the influence of lifestyle on bone remodelling should be assessed through long-term analyses [27].

One should mention the limitations to which this study was subject. Firstly, the presented material contains cross-sectional data, whereby the possibilities of drawing conclusions and explaining the causes of the investigated phenomena are limited. Secondly, bone density was measured in the region of the distal epiphyses of the forearm bones, which measurement is recommended mainly for screening tests. Nevertheless, the measurement results correlate with the measurements performed in the regions of the lumbar spine and the femoral bone neck. Thirdly, the study participants came mainly from the south-western Poland, but the number of participants—756 women—was relatively large.

## 5. Conclusion

As part of our study, 756 women aged 50-80 years were tested. The women who took part in this project were subjectively healthy, independent, and physically and socially active persons. They attended classes organized by senior citizen clubs and Universities of the Third Age.

The results of the tests indicate differences in the levels of muscle strength and BMD in the 50-year-old women with, respectively, the early and late menopause. The late-menopausal women score better results in motor ability and BMD tests. The differences decrease in the groups of 60-year-old women, while in the case of 70-year-old women, the results scored by, respectively, the early- and late-menopausal women are similar.

The rate of decline in hand grip strength, the functional efficiency of the lower and upper limbs, and BMD is faster in the late-menopausal women. For this reason, the test results for both the analysed groups of 70-year-old women are similar.

In the group of 50-year-old women, a higher percentage of persons with lowered bone mass occurs in the group of early-menopausal women. In the subsequent decades, the percentage of persons with a lowered bone mass increases in both groups, but to a larger extent in the late-menopausal women. Consequently, the percentage of persons characterized by a lowered bone mass in the groups of early- and late-menopausal 70-year-old women is similar.

Summing up, a higher percentage of persons with a lowered bone mass and a lower strength level was found in the group of early-menopausal women. Therefore, in the case of such women, proper prevention programmes should be launched as early as possible in order to minimize the risk of falls and osteoporotic fractures.

## Figures and Tables

**Table 1:** The statistical characteristic parameters of somatic features in the age groups of women with early menopause (<50 years old) and late (>50 years old).

Age (yrs)	50-59 (*n* = 166)				60-69 (*n* = 442)				70-79 (*n* = 148)			
Menopause	Early (n =90)		Late (n =76)		Early (n =216)		Late (n =226)		Early (n =86)		Late (*n* = 62)	
Variable	Mean	SD	Mean	SD	Mean	SD	Mean	SD	Mean	SD	Mean	SD
Height (cm)	161.30	6.02	161.74	5.97	159.12	5.49	158.99	5.82	157.56	5.29	155.24	5.79
Weigth (kg)	70.43	12.31	73.71	12.63	72.38	12.93	71.01	11.57	70.07	11.62	68.98	11.09
BMI	27.05	4.38	28.19	4.68	28.62	5.18	28.08	4.31	28.24	4.61	28.62	4.34

**Table 2:** The statistical characteristic of lifestyle factors in the age groups of women with early menopause (<50 years old) and late (>50 years old).

Age [yrs]		50-59 (*n* = 166)		60-69 (*n* = 442)		70-79 (*n* = 148)	
Menopause		Early (*n* = 90)	Late (*n* = 76)	Early (*n* = 216)	Late (*n* = 226)	Early (*n* = 86)	Late (*n* = 62)
		%	%	%	%	%	%
Smoke	Yes	20.2	10.5	9.4	8.4	5.2	4.8
	No	79.8	89.5	90.6	91.6	94.8	95.2
Alcohol	Yes	21.4	14.5	12.7	19.5	14.3	12.9
	No	78.6	85.5	87.3	80.5	85.7	87.1
Education	Lower	68.9	59.2	67.0	63.7	61.2	47.0
	Higher	11.1	40.8	33.0	36.3	38.8	53.0
Living	Alone	46.1	30.3	49.3	44.7	62.0	54.5
	Together	53.9	69.7	50.7	55.3	38.0	56.5

**Table 3:** Significance of differences between women with early and late menopause in age groups (Spearman rank-order).

Variables Age (yrs)	Early versus late					
	50-59		60-69		70-79	
	*F*	*p*	*F*	*p*	*F*	*p*
Smoke	-0.13	0.1063	-0.03	0.5581	-0.01	0.9247
Alcohol	-0.97	0.2140	0.04	0.4384	0.04	0.6473
Education	0.62	0.4237	0.08	0.1077	0.12	0.1621
Living	-0.15	0.0540	0.03	0.5024	0.11	0.1977

**Table 4:** The impact of age and time of menopause on the strength, BMD and somatic parameters – main effects of two - factor analysis of variance (significance of differences in bold p <0.05).

Variable	Factor					
	Age		Menopause		Interaction menopause x age	
	F	P	F	p	F	p
Hand grip	21.58	**0.0000**	0.21	0.6466	1.47	0.2317
Arm curl	15.17	**0.0000**	7.83	**0.0053**	3.70	**0.0251**
30-second chair stand	20.26	**0.0000**	4.78	**0.0291**	1.00	0.3697
Knee extensor muscle strength	19.87	**0.0000**	1.38	0.2400	4.11	**0.0168**
Knee flexor muscles strength	13.05	**0.0000**	0.57	0.4519	0.22	0.8014
BMD	62.70	**0.0000**	1.89	0.1697	1.90	0.1505
T – Ratio [%]	62.62	**0.0000**	2.66	0.1036	1.99	0.1368
Z – Ratio [%]	40.68	**0.0000**	2.31	0.1289	1.74	0.1764
Height	2.00	0.1553	3.10	**0.0000**	2.60	0.0724
Weight	0.07	0.7865	2.13	0.1198	2.27	0.1042
BMI	0.72	0.3958	1.68	0.1875	2.09	0.1247

**Table 5:** Detailed comparisons of means by LSD test (significance of differences in bold p <0.05).

Menopause	Early versus late			Early		Late	
Age [yrs] Variable	50-59	60-69	70-79	50 versus 60	60 versus 70	50 versus 60	60 versus 70
Hand grip	0.7113	0.4412	0.1375	**0.0011**	0.1804	**0.0026**	**0.0004**
Arm curl	**0.0064**	**0.0001**	0.5046	0.3075	0.1329	0.1591	**0.0000**
30-second chair stand	**0.0275**	0.0869	0.8603	0.2297	**0.0025**	**0.0116**	**0.0001**
Knee extensor muscle strength	**0.0062**	0.4217	0.1873	0.6185	**0.0030**	**0.0287**	**0.0000**
Knee flexor muscles strength	0.3634	0.6328	0.9874	0.5312	**0.0006**	0.8964	**0.0003**
BMD	**0.0463**	0.1155	0.4497	**0.0001**	**0.0000**	**0.0000**	**0.0000**
T – Ratio [%]	**0.0316**	0.0683	0.5039	**0.0001**	**0.0001**	**0.0000**	**0.0000**
Z – Ratio [%]	**0.0466**	0.0819	0.5279	**0.0042**	**0.0006**	**0.0001**	**0.0000**
Height	0.6216	0.8114	**0.0140**	**0.0029**	**0.0374**	**0.0000**	**0.0000**
Weigth	0.0858	0.2375	**0.0197**	0.2077	0.1471	0.0936	0.2270
BMI	0.1189	0.2221	0.6203	**0.0080**	0.5294	0.8598	0.4016

**Table 6:** The statistical characteristic parameters of strength and bone mineral density in the age groups of women with early menopause (<50 years old) and late (>50 years old).

Age (yrs)	50-59 (*n* = 166)				60-69 (*n* = 442)				70-79 (*n* = 148)			
Menopause	Early (*n* = 90)		Late (*n* = 76)		Early (*n* = 216)		Late (*n* = 226)		Early (*n* = 86)		Late (*n* = 62)	
Variable	Mean	SD	Mean	SD	Mean	SD	Mean	SD	Mean	SD	Mean	SD
Hand grip (kg)	28.81	7.53	29.17	6.53	26.18	5.67	26.65	6.20	25.08	6.96	23.53	5.71
Arm curl (*n*)	20.43	4.01	22.34	5.10	19.84	4.31	21.51	4.43	18.96	4.84	18.47	4.37
30-second chair stand (*n*)	17.59	3.88	19.00	4.80	16.96	4.17	17.63	4.00	15.34	3.70	15.46	3.82
Knee extensor muscle strength (N/m)	196.15	79.51	231.18	94.91	201.30	81.92	207.55	82.37	169.55	78.54	151.87	61.56
Knee flexor muscles strength (N/m)	97.24	46.11	103.18	41.47	100.56	40.52	102.46	41.25	81.70	41.78	81.59	39.86
BMD (g × cm^–2^)	0.38	0.08	0.40	0.08	0.34	0.09	0.35	0.07	0.29	0.07	0.28	0.08
*T*-ratio (%)	76.66	15.06	81.89	15.47	68.71	16.62	71.41	14.83	60.44	14.53	58.73	15.23
*Z*-ratio (%)	95.39	18.94	101.66	19.31	88.06	21.28	91.40	19.21	79.04	19.62	76.95	21.34

**Table 7:** The percentage of women with lowered bone mass by age and time of menopause.

Menopause	Age [years]		
	50-59 (n =166)	60-69 (n =442)	70-79 (n =148)
Early [%]	36.7 (n =90)	52.1 (n =216)	73.8 (n =86)
Late [%]	21.1 (n =76)	43.7 (n =226)	72.1 (n =62)

## Data Availability

Data are available upon request due to ethical restrictions regarding participant privacy. Requests for the data may be sent to the corresponding author.
